# Case report: Clinical and pathological findings in a canine patient with intervertebral disk extrusion resembling progressive myelomalacia

**DOI:** 10.3389/fvets.2023.1122566

**Published:** 2023-03-16

**Authors:** Annie Lin, Rachel Lampe, Carsten Bandt, Miranda Vieson, Jae Yoon Park

**Affiliations:** ^1^Department of Emergency and Critical Care, Neurology, and Diagnostic Imaging, Canada West Veterinary Specialists and Critical Care Hospital, Vancouver, BC, Canada; ^2^Department of Veterinary Clinical Medicine, University of Illinois at Urbana-Champaign, Urbana, IL, United States

**Keywords:** myelomalacia, IVDE, cervical disc extrusions, ventral slot, mechanical ventilation

## Abstract

A 4-year-old female spayed dog presented to the emergency department for non-ambulatory tetraparesis, which progressed to tetraplegia. Computed tomography (CT) confirmed cervical intervertebral disk extrusion at C5-6 extending to C6-7, and an emergency ventral slot was performed. After the procedure, the patient was placed on mechanical ventilation due to respiratory failure. Repeat assessment upon weaning her ventilatory support suggested the patient's neurological status had declined. Based on her deterioration and suspicion of progressive myelomalacia on magnetic resonance imaging (MRI), she was euthanized. Post-mortem histopathology of the spinal cord supported the presence of progressive myelomalacia. To the author's knowledge, this is the first case report describing a progressive myelomalacia in a canine patient with cervical intervertebral disk extrusion.

## Introduction

Cervical intervertebral disk extrusion (IVDE) is generally considered to have a good prognosis, with upwards of 95% improvement following decompressive surgery even when complications occur (1–3). Serious adverse effects related to ventral slot surgery are rare, occurring in only 6.4% of cases in one large study, and are rarely fatal (1). Progressive myelomalacia is a fatal progressive disease process that is most commonly documented in dogs secondary to an acute intervertebral disk extrusion in the thoracolumbar spine (4, 5). It has been previously described as progressive ascending and/or descending hemorrhagic necrosis subsequent to acute IVDE (4, 5).

Risk factors for progressive myelomalacia after IVDE have been identified to include the severity of the injury, age, site of disk herniation, and becoming non-ambulatory within 24 h of developing clinical signs (6). Unfortunately, no treatment is currently available; therefore, patients are humanely euthanized after the onset of progressive myelomalacia or die of respiratory failure (7–9). While some studies have suggested that the odds of developing progressive myelomalacia may be decreased through factors such as faster surgical intervention, performing extensive decompression (in comparison to hemilaminectomy alone), and use of corticosteroids, generally there is no way to predict which dogs will develop the condition before surgery (10, 11). The diagnosis of progressive myelomalacia relies on a combination of neurological exam findings, magnetic resonance imaging, cerebral spinal fluid (CSF) analysis, biomarkers, and/or histopathology (7, 12–17). Several diagnostic biomarkers for IVDE have been previously investigated as outcome predictors (14–16).

Previously reported cases of progressive myelomalacia in veterinary medicine have been only associated with IVDE in the thoracolumbar spinal cord and infrequently reported in patients with fibrocartilaginous embolism (13). To the author's knowledge, there has been no previous literature on cervical IVDE developing progressive myelomalacia.

## Case description

A 4-year-old female spayed, 10 kg non-chondrodystrophic mixed breed dog was presented to the emergency department for acute tetraparesis. The patient was rescued from Mexico in 2018, and other than diet-related allergies and suspected left luxating patella, she was considered to be overall healthy. Clinical signs started 2 days prior to the presentation including soft stool, lethargy, and hyporexia. The patient first presented to her primary care veterinarian for the listed symptoms and development of a stiff pelvic limb gait.

Complete blood cell count, biochemistry, canine pancreas-specific lipase, and urine drug screen were performed when she was evaluated by her primary care veterinarian. Mild thrombocytopenia was reported at 119 × 10^9^/L (range 148–484 × 10^9^/L), and she was noted to be trace positive for tricyclic antidepressants on the urine drug screen. During her visit, hindlimb rigidity developed. She received intravenous (IV) maropitant (1 mg/kg), lactated Ringer's solution (100 ml/h), and was fed 23 ml of activated charcoal. Two hours after presentation, her pelvic limb rigidity was reported to have worsened, and she received a dose of 0.5 mg/kg diazepam IV for a possible seizure prior to arranging for a direct transfer to our specialty hospital.

On presentation to the emergency department, she was tachycardic but otherwise cardiovascularly stable. She was evaluated to be subjectively non-painful (Colorado pain score 0/4). The neurological evaluation demonstrated slightly depressed mentation (suspected secondary to recent administration of diazepam). Cranial nerve examination was unremarkable, but due to aggression, she did not tolerate assessment of physiological nystagmus. She was non-ambulatory tetraplegic with exaggerated spinal reflexes in her pelvic limbs and reduced withdrawals of her thoracic limbs. Pain sensation was present on all four limbs, and spinal pain was not elicited on admission. Her examination was consistent with a C1-T2 myelopathy.

Point of care blood work on admission included packed cell volume/total solids, venous blood gas, electrolytes, metabolites, and co-oximetry, which were all within normal limits. She received isotonic crystalloid IV fluid therapy at 20 ml/h with potassium chloride supplementation at 20 mEq/L and was hospitalized overnight. Blood gas analysis was performed 6 h after admission. Ionized hypercalcemia was identified (1.88 mmol/L, range 1.2–1.5mmol/L) with the remaining parameters within normal limits. Overall, her neurological status was stable overnight, and she was transferred to the neurology service in preparation for further diagnostic workup.

**Figure 1:**
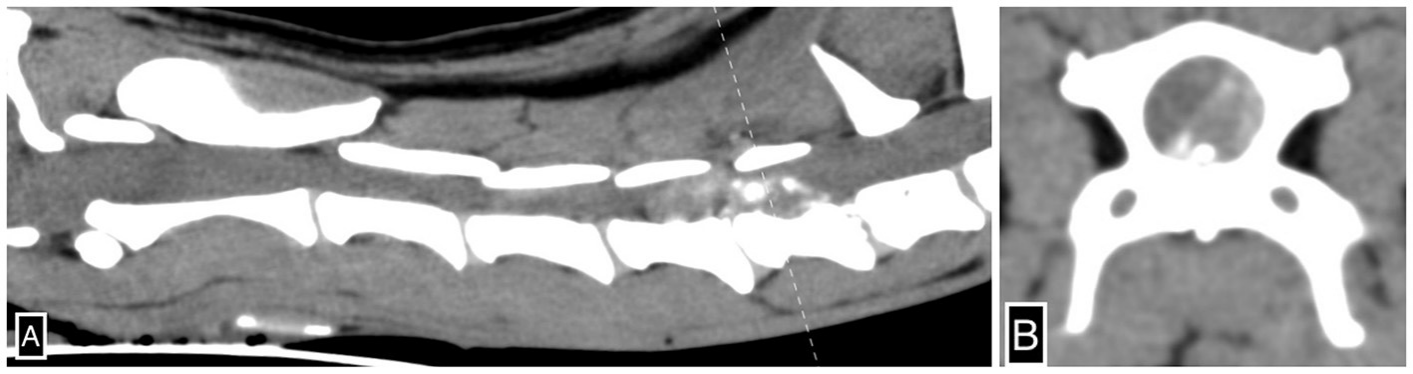
Severe left-sided C5-6 intervertebral disk extrusion. Sagittal **(A)** and transverse **(B)** CT images of the cervical spine illustrate severe mineral-attenuating extradural material in the left ventral aspect of the vertebral canal centered at C5-6 extending cranially to mid-C5 and caudally to C6-7 and subsequent extradural spinal cord compression. The dotted line **(A)** denotes the axis by which the transverse CT image **(B)** is reconstructed.

The next morning she became hypothermic (33.3°C) and mildly hypotensive with oscillometric systolic blood pressure at 102 mmHg. Hypoventilation along with poor thoracic movements during inspiration was noted. Hemoglobin oxygen saturation (SPO_2_) was 99% while breathing room air. Repeat blood gas showed the development of hypercapnia with a partial pressure of carbon dioxide in venous blood of 67.3 mmHg (range 37–45 mmHg), with normal electrolyte parameters. Her neurological examination demonstrated stuporous mentation along with decreased menace bilaterally and poor oculocephalic response. She had absent withdrawal reflexes in her thoracic limbs with withdrawal reflexes intact in her pelvic limbs, and she remained tetraplegic. She had an absent cutaneous trunci reflex, and no spinal pain was elicited. Her nociception was examined and was absent in all four limbs. She received 20 ml of 7.2% hypertonic saline IV, with no significant improvement in mentation. Due to severe hypoventilation and the development of cyanosis, she received alfaxalone for intubation (size 7 endotracheal tube) and manual ventilation. An arterial blood gas was obtained an hour after intubation demonstrating respiratory alkalosis (pH 7.518, range 7.32–7.43; CO_2_ 28.3 mmHg, range 37–45 mmHg). Given her acute deterioration, the patient underwent CT (Toshiba Aquilion 64; Tokyo; Japan) of her head and cervical spine. No contrast was administered. She received IV fentanyl citrate (2.5 μg/kg) and midazolam (0.2 mg/kg) and was supported with ongoing manual positive pressure ventilation. A measure of 0.5 mm transverse axial CT images of the head and the neck was reconstructed from a volume acquired *via* a helical scan with an index of 0.5 mm. This CT study demonstrated a severe amount of mineral attenuating extradural material in the left ventral aspect of the vertebral canal extending from the level of mid C5 to C6-7, causing severe rightward dorsal displacement and extradural compression of the spinal cord, suggestive of severe left-sided C5-6 intervertebral disk extrusion (Figure 1). The maximum compression of the spinal cord was noted over the body of C6, and the mineral attenuating extradural material occupied up to 70% of the vertebral canal lumen. The rest of the scan was considered within normal limits. Subsequent to the CT scan, she received one injection of 0.1 mg/kg dexamethasone IV and underwent a ventral slot procedure at C5-6 and C6-7 removing 50–75% of the disk material, as confirmed on postoperative CT. Anesthesia protocol included lidocaine CRI (constant rate infusion, 50 μg/kg/min), ketamine CRI (5 μg/kg/min), fentanyl CRI (15 μg/kg), and isoflurane (minimum alveolar concentration 0.75–1%). Postoperative analgesia was provided with fentanyl (3 μg/kg/h), ketamine (2.5 μg/kg/min), and lidocaine (25 mg/kg/min). On recovery, persistent hypoventilation with minimal chest excursion was noted. Arterial blood gas showed the development of hypercapnia at 70.4 mmHg. The patient could not be safely extubated given respiratory failure and hypercapnia; therefore, mechanical ventilation was initiated (Dräger, Evita 4 edition; Mississauga, ON) on mandatory minute ventilation mode (Fraction of inspired oxygen 0.3; tidal volume 9 ml/kg; respiratory rate 36/min; inspiratory to expiratory ratio 1:1.6; positive end-expiratory pressure 5 cm H_2_O).

Total intravenous anesthesia (TIVA) was achieved using fentanyl (8–10 μg/kg/h), midazolam (0.2 mg/kg/h), ketamine (2.5–5 μg/kg/min), and propofol (60–90 μg/kg/min) CRI. The patient developed mild systolic hypotension (95 mmHg) 4 h after initiating mechanical ventilation and was supported with a dopamine CRI (5–7 μg/kg/min). Oliguria was noted (urine output 0.87–1.21 ml/kg/h).

The owners elected to attempt to wean her off the mechanical ventilator after 24 h of respiratory support, to re-evaluate whether she had regained her ventilator drive. Upon weaning her TIVA, the patient began masticating her endotracheal tube. She was extubated but was promptly re-intubated, as she became cyanotic. No spontaneous breathing or abdominal excursions were witnessed post-extubation. Upon repeat neurological evaluation, she had persistent absent nociception on all four limbs and tails. She was mentally obtunded despite discontinuing most of her TIVA except for low-dose ketamine CRI at 2 μg/kg/min.

**Figure 2:**
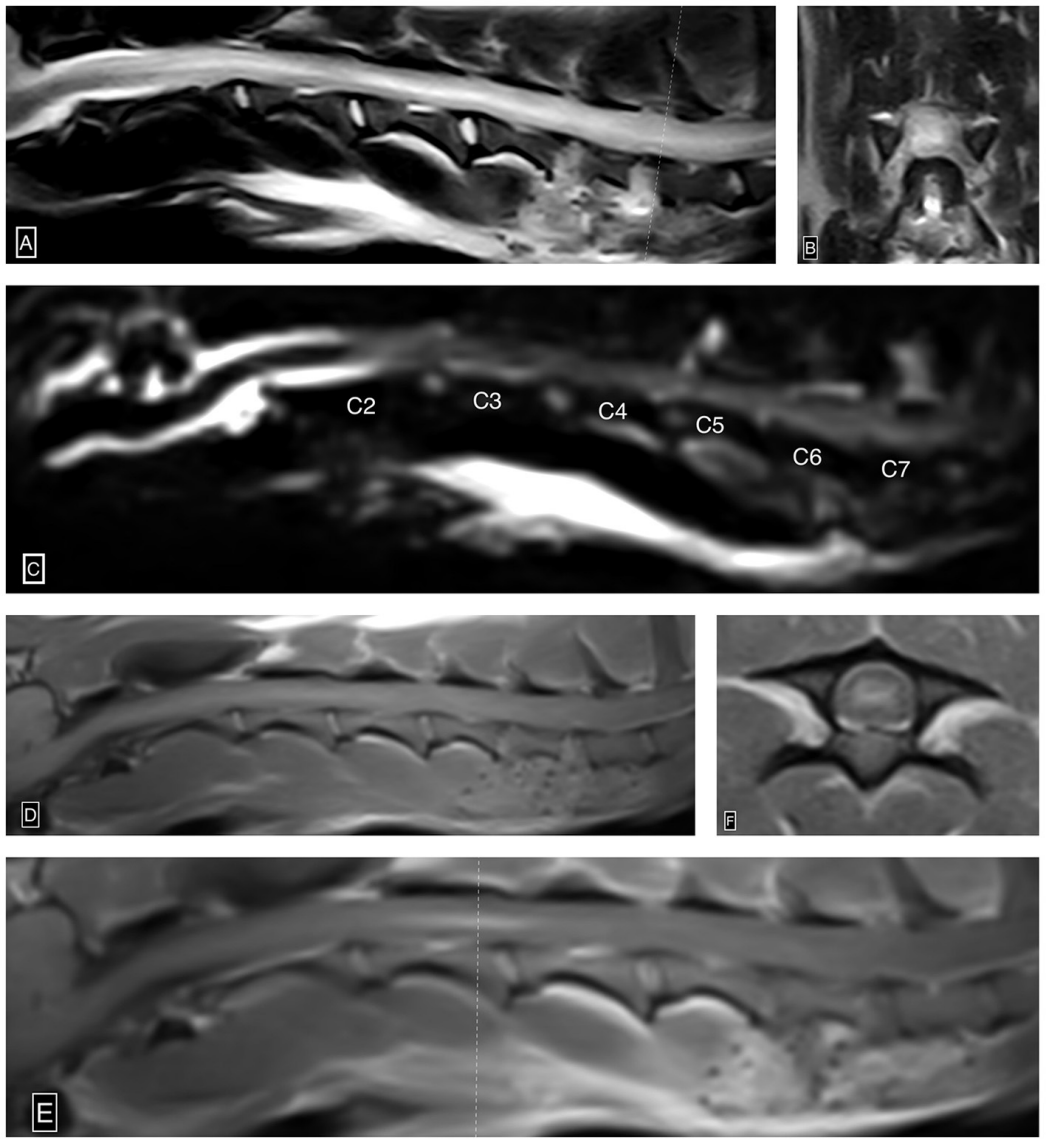
Ascending/descending cervical myelomalacia. Sagittal **(A)** and transverse **(B)** T2W images show severe diffusely and heterogeneously increased intramedullary T2W signal intensity within the cervical spinal cord centered over the gray matter. Notably, the recent C5-6 and C6-7 hemilaminectomy sites characterized by ostectomy and local soft tissue thickening and increased signal intensity **(A, B)**. Sagittal HASTE image **(C)** shows complete and circumferential attenuation of the cervical subarachnoid space from the level of mid-C2 beyond the caudal limit of the study (level of T2), suggestive of extensive spinal cord swelling. Pre-contrast sagittal T1W **(D)**, post-contrast sagittal T1W **(E)**, and post-contrast transverse T1W **(F)** images demonstrate ill-defined and heterogeneous intraparenchymal contrast enhancement centered over the gray matter from the level of C2-3 through mid C4. The dotted line **(E)** denotes the axis by which the post-contrast T1W image is acquired.

The patient subsequently underwent an MRI (Esaote MR Vet Grande 0.25 tesla; Geneva; Italy) of the neck. Transverse and sagittal MRI images of the neck were acquired in T2-weighted (T2W), T1-weighted (T1W), T2W^*^, and fluid-attenuated inversion recovery (FLAIR) sequences pre- and post-gadolinium administration. This study demonstrated severe diffusely and heterogeneously increased intramedullary T2W signal intensity within the spinal cord parenchyma centered over the gray matter extending from the level of C1-2 caudally beyond the caudal limit of the cervical spine study at the level of T2 (approximately over 18 cm, nine times the C2 vertebral length). This abnormal cervical intramedullary hyperintensity caused near complete obliteration of the gray matter to white matter distinction and central canal conspicuity. Within the spinal cord, gray matter extending from the level of C2-3 through C4, there was moderate ill-defined and heterogeneous intraparenchymal contrast enhancement. There was complete and circumferential attenuation of the subarachnoid space extending from the level of mid-C2 caudally beyond the caudal limit of the cervical spine study (level of T2), consistent with severe spinal cord swelling. There was no evidence of a T2W^*^susceptibility artifact identified in the study. These MRI findings were consistent with imaging findings associated with progressive myelomalacia in the thoracolumbar spine, as well as ischemic necrosis (Figure 2). At this point, given the primary suspicion of progressive myelomalacia along with the grave prognosis, the owners opted for euthanasia. A measure of 11 ml of IV pentobarbital was administered, and cardiac arrest was confirmed with auscultation. The brain and cervical spinal cord to the level of T1 were submitted for histopathology interpretation.

**Figure 3:**
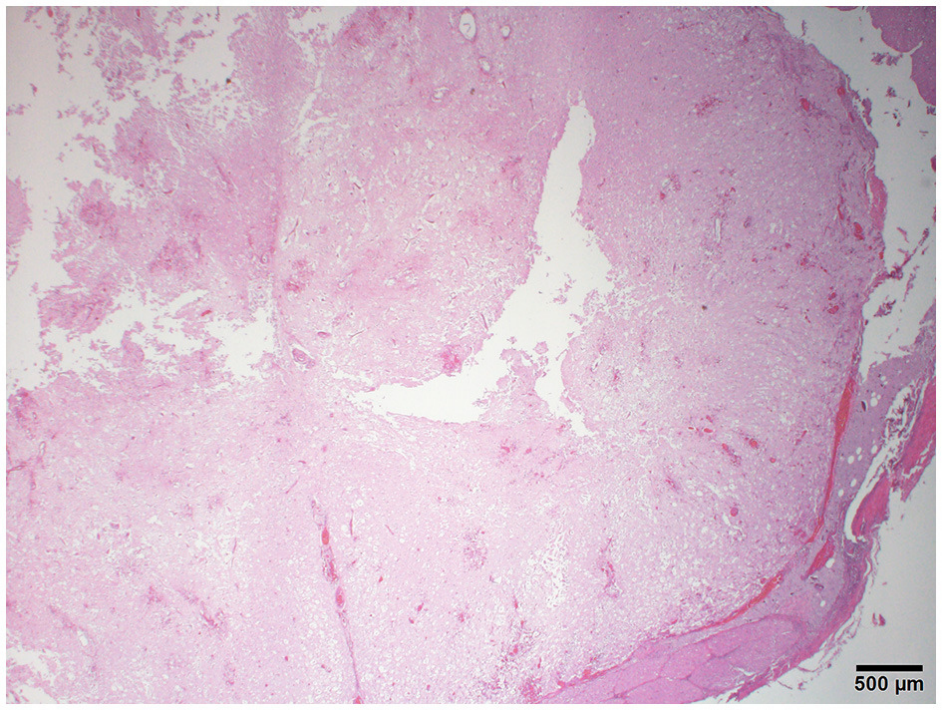
Photomicrograph of the cervical spinal cord with multifocal hemorrhage, severe tissue loss, cavitation, and lack of distinction between the gray and white matter. H&E stain.

**Figure 4:**
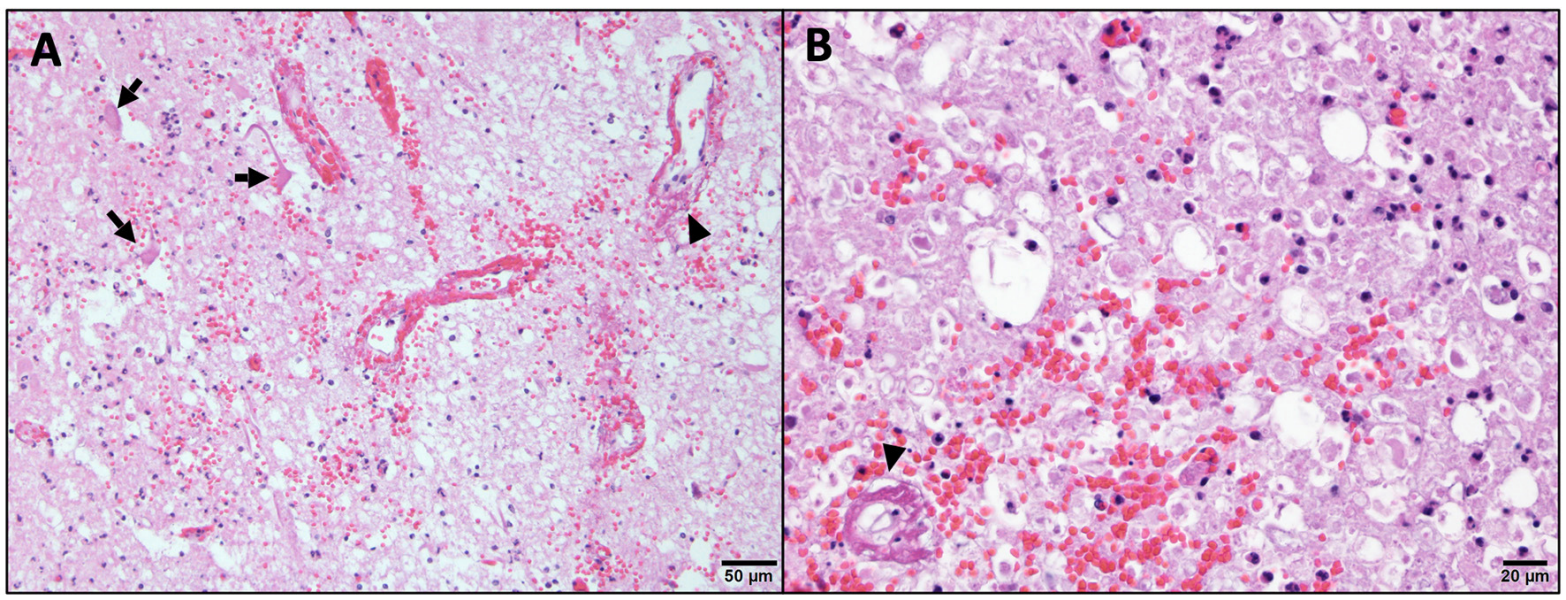
Higher magnification of microscopic changes in the cervical spinal cord. Within the gray matter **(A)**, there is neuropil rarefaction and vacuolation with low numbers of residual neurons being angular and hypereosinophilic with no nucleus (arrow, necrosis). Within the white matter **(B)**, there is swelling and hypereosinophilia of axons and marked distension of myelin sheaths, some of which lack axons (axonal loss). In both the gray and white matter, there is multifocal hemorrhage, scattered infiltration by low numbers of neutrophils, and multiple vessel walls are partial to fully obscured by bright eosinophilic fibrillary material (arrow head, fibrinoid vascular necrosis). H&E stain.

The histopathology of her spine showed changes proximal to IVDE sites and was supportive of hemorrhagic progressive myelomalacia. The spinal cord had a loss of distinction between the white and gray matter. This was attributed to acute necrosis, multifocal to coalescing hemorrhage, loss of tissue with occasional cavitation, and mild neutrophilic inflammation (Figure 3). Within and around areas of necrosis, multiple thin-walled vessels were lined with decreased numbers of reactive endothelial cells and had fibrinoid vascular necrosis. In some less severely affected spinal sections, the residual white matter was often comprised of multiple swollen axon sheaths containing swollen hypereosinophilic and glassy axons (spheroids) and rare macrophages (digestion chambers) or was empty from axonal dropout (Figure 4). Histopathology of the brain had mild perivascular cuffs of lymphocytes, plasma cells, and rare macrophages in the meninges and choroid plexus consistent with mild inflammation.

## Discussion

Progressive myelomalacia is well documented in dogs secondary to severe thoracolumbar IVDE. However, it has not previously been reported secondary to cervical disk disease. Although the basic physiology of disk extrusions in the cervical and thoracolumbar spine is similar, there are several clinical and histopathological differences. The pathophysiology of progressive myelomalacia is not well understood, although impairment of vascular perfusion secondary to mechanical impact from IVDE is suspected to contribute a significant role following ischemia and accumulation of free radicals (6, 8, 12). One study comparing the epidural pathology of material removed during spinal decompressive surgery found that cervical IVDE was associated with a less intense inflammatory reaction, which may help explain why this complication has not been reported in the cervical disk (18).

Progressive myelomalacia most commonly affects dogs with absent deep pain perception. Well-known risk factors for its development in the thoracolumbar spine include the severity of neurology signs (i.e., becoming paraplegic with absent nociception) and a rapid progression of clinical signs (6). This patient had both of these risk factors, with loss of mobility within 24 h, and being tetraplegic without pain sensation; thus, cervical IVDE may share some of the same risk factors.

Since patients who lose pain sensation due to cervical myelopathies are at risk of respiratory musculature and diaphragm paralysis, patients are likely to die (or be euthanized) before imaging and surgery. This means that patients with severe cervical IVDE are likely euthanized before there has been time to develop progressive myelomalacia, unlike in thoracolumbar patients who may be cardiopulmonary stable despite being paralyzed. Patients who are tetraplegic are also likely to require ventilation, thus adding significant financial burden, and are subsequently more likely to be euthanized without treatment. A previous retrospective study reported 4.9% of canine patients with cervical lesions post-ventral slot to require mechanical ventilatory support secondary to hypoventilation (19). Although the study reported a good prognosis after carrying out ventilatory support, the presence of progressive myelomalacia would significantly change our patients' outcomes, making it important to recognize. Furthermore, the requirement of general anesthesia for mechanical ventilation would likely prevent complete neurological evaluation, thus masking early signs of progressive myelomalacia without weaning the patient off of TIVA.

The diagnosis of progressive myelomalacia can be difficult to confirm antemortem, but clinical suspicion can be supported by MRI, CSF cytology, and/or biomarkers. MRI changes in thoracolumbar IVDE cases that have been associated with the development of progressive myelomalacia include the length of attenuation of the half-Fourier single-shot turbo spin-echo (HASTE) signal and intramedullary T2 hyperintensity, both as a ratio to L2 length (7). In this case report, the extensive HASTE attenuation and T2 intramedullary hyperintensity both extended at least six intervertebral disk spaces and extended past the caudal end of the imaging study. An extensive T2 intramedullary hyperintensity and/or significant HASTE attenuation should both raise the index of suspicion for progressive myelomalacia in dogs with IVDE in the cervical spine. CSF findings including neutrophilic pleocytosis, high total nucleated cell count, and high protein concentration may be supportive of progressive myelomalacia, although they are non-specific and have been detected in CSF samples, with acute IVDE in patients that make a good recovery (12, 20). The utility of biomarkers remains limited to research settings and is not widely available in commercial laboratories, although recent literature investigating glial fibrillary acidic protein (GFAP) and serum phosphorylated neurofilament-heavy chain (pNF-H) has shown promising results in detecting progressive myelomalacia in thoracolumbar IVDE (14–16). Given the limitations surrounding antemortem progressive myelomalacia diagnosis and the inability to monitor a ventilated/anesthetized patient's neurological status, detecting progressive myelomalacia secondary to cervical IVDE poses a significant challenge. While MRI is the most useful diagnostic test at this time, further evaluation of biomarkers may be useful in guiding the clinical outcome of our patients and allow clients to make a well-informed decision in terms of whether to proceed with surgery and mechanical ventilation, respectively. The limitations of this case report include the single sample size given the novelty of our finding and the fact the patient's thoracolumbar spinal cord was not imaged or submitted for histopathology review to determine the extent of progressive myelomalacia. CSF samples should also be considered in the future for cytological interpretation and to look into GFP and pNF-H levels.

In conclusion, this case report highlights the first-ever case of cervical IVDE resembling the progression of myelomalacia and should be discussed as a possible outcome/complication in patients undergoing a ventral slot.

## Data availability statement

The original contributions presented in the study are included in the article/supplementary material, further inquiries can be directed to the corresponding author.

## Ethics statement

Ethical review and approval was not required for the study on animals in accordance with the local legislation and institutional requirements. Written informed consent was obtained from the owners for the participation of their animals in this study.

## Author contributions

AL managed mechanical ventilation and created and finalized the manuscript. RL performed the neurological examination and ventral slot surgery and assisted in multiple draft revisions. CB assisted in multiple draft revisions. MV interpreted the results of histopathology and provided histopathology images. JP interpreted the findings of diagnostic imaging and provided imaging figures. All authors contributed to the manuscript and approved the submitted version of the manuscript.

## References

[1] Rossmeisl JH White C Pancotto TE Bays A Henao-Guerrero PN. Acute adverse events associated with ventral slot decompression in 546 dogs with cervical intervertebral disc disease. Vet Surg. (2013) 42:795–806. 10.1111/j.1532-950X.2013.12039.x 23980621

[2] Brisson BA. Intervertebral disc disease in dogs. Vet Clin North Am Small Anim Pract. (2010) 40:829–58. 10.1016/j.cvsm.2010.06.001 20732594

[3] Schmied O Golini L Steffen F. Effectiveness of cervical hemilaminectomy in canine Hansen Type I and Type II disc disease: a retrospective study. J Am Anim Hosp Assoc. (2011) 47:342–50. 10.5326/JAAHA-MS-5604 21852506

[4] Sharp NJH Wheeler SJ. Small Animal Spinal Disorders. 2nd ed. New York, NY: Elsevier (2005). 10.1016/B978-0-7234-3209-8.X5001-8

[5] Griffiths IR. The extensive myelopathy of intervertebral disc protrusions in dogs ('the ascending syndrome'). J Small Anim Pract. (1972) 13:425–38. 10.1111/j.1748-5827.1972.tb06870.x 5081200

[6] Balducci F Canal S Contiero B Bernardini M. Prevalence and risk factors for presumptive ascending/descending myelomalacia in dogs after thoracolumbar intervertebral disk herniation. J Vet Intern Med. (2017) 31:498–504. 10.1111/jvim.14656 28144987 PMC5354033

[7] Castel A Olby NJ Mariani CL Muñana KR Early PJ. Clinical characteristics of dogs with progressive myelomalacia following acute intervertebral disc extrusion. J Vet Intern Med. (2017) 31:1782–9. 10.1111/jvim.14829 28961348 PMC5697170

[8] de Lahunta A Glass EM Kent M. Veterinary Neuroanatomy and Clinical Neurology. 4th ed. Missouri: Elsevier (2020). p. 146–7.

[9] Sharp NJ Wheeler SJ. “*Thoracolumbar Disc Disease” Small Animal Spinal Disorders*. Edinburgh: Elsevier (2005). p. 121–59.

[10] Castel A Olby NJ Ru H Mariani CL Muñana KR Early PJ. Risk factors associated with progressive myelomalacia in dogs with complete sensorimotor loss following intervertebral disc extrusion: a retrospective case-control study. BMC Vet Res. (2019) 15:1–9. 10.1186/s12917-019-2186-0 31796017 PMC6892155

[11] Nakamoto Y Uemura T Hasegawa H Nakamoto M Ozawa T. Outcomes of dogs with progressive myelomalacia treated with hemilaminectomy or with extensive hemilaminectomy and durotomy. Vet Surg. (2021) 50:81–8. 10.1111/vsu.13514 33280138

[12] Midori Okada D Masato Kitagawa DP Daisuke Ito DP Takuya Itou DP Kiichi Kanayama DVMP. Magnetic resonance imaging features and clinical signs associated with presumptive and confirmed progressive myelomalacia in dogs 12 cases (1997–2008). JAVMA. (2010) 237:1160–5. 10.2460/javma.237.10.1160 21073387

[13] Lu D Lamb CR Tarcett MP. Results of myelography in seven dogs with myelomalacia. Vet Radiol Ultrasound. (2002) 43:326–30. 10.1111/j.1740-8261.2002.tb01012.x 12174994

[14] Sato Y Shimamura S Mashita T Kobayashi S Okamura Y Katayama M. Serum glial fibrillary acidic protein as a diagnostic biomarker in dogs with progressive myelomalacia. J Vet Med Sci. (2013) 75:949–53. 10.1292/jvms.12-0483 23470323

[15] Olby NJ Lim JH Wagner N Zidan N Early PJ Mariani CL. Time course and prognostic value of serum GFAP, pNFH, and S100β concentrations in dogs with complete spinal cord injury because of intervertebral disc extrusion. J Vet Intern Med. (2019) 33:726–34. 10.1111/jvim.15439 30758078 PMC6430936

[16] Murthy VD Li CF Hicks J Kroll J Giuffrida M Dickinson P Toedebusch CM. Serum phosphorylated neurofilament heavy chain as a diagnostic biomarker for progressive myelomalacia in dogs with thoracolumbar intervertebral disc herniation. J Vet Intern Med. (2021) 35:2366–73. 10.1111/jvim.16251 34476832 PMC8478056

[17] Marquis A Packer RA Borgens RB Duerstock BS. Increase in oxidative stress biomarkers in dogs with ascending–descending myelomalacia following spinal cord injury. J Neurol Sci. (2015) 353:63–9. 10.1016/j.jns.2015.04.003 25912174

[18] Züger L Fadda A Oevermann A Forterre F Vandevelde M Henke D. Differences in epidural pathology between cervical and thoracolumbar intervertebral disk extrusions in dogs. J Vet Intern Med. (2018) 32:305–13. 10.1111/jvim.14887 29194770 PMC5787202

[19] Matthew W Beal DD Danielle T Paglia B Greg M Griffin MMD. Ventilatory failure, ventilator management, and outcome in dogs with cervical spinal_disorders_14 cases (1991–1999). JAVMA. (2001) 218:1598–1602. 10.2460/javma.2001.218.1598 11393372

[20] Windsor RC Vernau KM Sturges BK Kass PH Vernau W. Lumbar cerebrospinal fluid in dogs with type I intervertebral disc herniation. J Vet Intern Med. (2008) 22:954–60. 10.1111/j.1939-1676.2008.0141.x 18647156

